# Daily Monitoring of Behavioral and Psychological Symptoms of Dementia in Residential Care: Mixed Methods Pilot Study

**DOI:** 10.2196/98024

**Published:** 2026-07-23

**Authors:** Johannes Malm, Therése Bielsten, Charlotta Nilsen, Elzana Odzakovic, Ingemar Kåreholt

**Affiliations:** 1 Institute of Gerontology, School of Health and Welfare, Jönköping University, Barnarpsgatan 39, Box 1026, Jönköping, 551 11, Sweden, +46 70 764 71 73; 2 Department of Health, Blekinge Institute of Technology, Karlskrona, Sweden; 3 Aging Research Center, Karolinska Institutet, Stockholm, Sweden; 4 Department of Psychology, The Stress Research Institute, Stockholm University, Stockholm, Sweden; 5 Department of Nursing, School of Health and Welfare, Jönköping University, Jönköping, Sweden; 6 Division of Clinical Geriatrics, Department of Neurobiology, Care Sciences and Society (NVS), Karolinska Institutet, Stockholm, Sweden

**Keywords:** behavioral and psychological symptoms of dementia, BPSD, dementia care, digital health, high-frequency observational data, mixed methods research, Neuropsychiatric Inventory, NPI, pilot study, residential care

## Abstract

**Background:**

Many clinically relevant aspects of health are expressed through behavior, affect, and interaction, and therefore rely on observation rather than instrumental measurement; yet such information is often documented infrequently in routine care. Dementia, and particularly behavioral and psychological symptoms of dementia (BPSD), is a relevant case because symptoms fluctuate across time and context. Little is known about how structured daily symptom assessment can be integrated into routine dementia care practice.

**Objective:**

This pilot study aimed to investigate whether structured daily observation of BPSD, supported by digital documentation, can be incorporated into everyday residential dementia care.

**Methods:**

A convergent mixed methods design was applied across 4 nursing home units in southern Sweden. Feasibility was operationalized through 4 focus areas (implementation, practicality, acceptability, and integration), with adherence to the registration routine as the primary feasibility outcome. Quantitative data comprised structured registrations collected daily by care staff over 90 days for 8 residents with BPSD, analyzed descriptively, and interpreted using selected NASSS (nonadoption, abandonment, scale-up, spread, and sustainability) framework domains to contextualize variation in symptom patterns, adherence, and registration practices. Semistructured dyadic interviews were analyzed using deductive content analysis guided by the same domains, with integration occurring at the analytical and interpretive stages.

**Results:**

A total of 21,993 item-level registrations were completed: 19,123 NPI-NH (Neuropsychiatric Inventory – Nursing Home version) symptom ratings, recorded across 1623 shift registrations (1 per resident per shift), and 2870 individually selected variables. Overall adherence to the registration routine averaged 75% across shifts but varied considerably between residents (range 33%‐90%) and between staff members. Mean time per shift registration decreased from 77.1 seconds in the initial 30-day period to 46.2 seconds in the final 30-day period (*P*=.008). Symptom prevalence varied widely across NPI domains (7.5% to 38.1% of observation days), and daily symptom counts varied both between and within residents. The routine was experienced as technically straightforward and easy to incorporate into daily work. At the same time, challenges emerged, including time constraints, inconsistent participation, varying levels of interpretive confidence, and difficulties distinguishing among overlapping symptoms. Organizational routines and team stability were central to sustaining staff engagement.

**Conclusions:**

The high number of completed registrations indicates that daily observations can be integrated into daily dementia care. The participants perceived it as meaningful and useful. Feasibility was primarily influenced by organizational and interpretive factors rather than technical constraints. Further research should explore strategies to support shared routines and sustain engagement over time.

## Introduction

### Care Under Pressure and the Promise of Digitalization

Health care systems in many countries, including Sweden, are under pressure from demographic change: an increasing number of older adults with chronic conditions and complex care needs are met by limited staff and resources [1-4]. At the same time, care is expected to be evidence-based and person-centered [5,6], a standard that is difficult to meet in everyday practice characterized by high workload, time pressure, and gaps in clinical information sharing [7,8]. Digitalization is often proposed as one means to address this gap, through documentation, data collection, and information sharing that can strengthen the informational basis for clinical decision-making and enable more detailed, frequent data on patients’ health status [9-11].

Digitalization has expanded the possibility of collecting high-frequency health data that capture patterns and temporal dynamics often missed by episodic assessment and documentation. In several areas of medicine, increasing the frequency of data collection has yielded more detailed insight into health over time, for example, through continuous glucose monitoring in diabetes care [12] and data-intensive cancer diagnostics [13,14]. These advances, however, rely on data generated by sensors or imaging. Many clinically important phenomena, including behavioral and psychological symptoms of dementia (BPSD), are instead captured through staff observation and subjective interpretation, where structured, frequent data are far less available and data-driven methods correspondingly less established. Realizing comparable benefits in such domains therefore depends first on whether observational data can be captured systematically and frequently enough in everyday practice.

### The Documentation Gap in Observational Care

In such observation-based aspects of care, professional judgment remains central to detection and interpretation [15], and both the assessment routine itself and the data it generates can provide important insight into a person’s situation, needs, and well-being [16]. However, these assessments are typically documented in episodic, narrative formats that lack the standardization and temporal resolution needed to detect gradual or subtle changes over time [17]. This stands in contrast to instrumental measurements, where increasingly continuous and automated data collection enables fine-grained tracking of change [18].

This documentation gap is particularly evident in long-term care settings, where individuals rely on staff to observe and interpret their needs. Through continuous interaction, staff accumulate substantial tacit knowledge about behavioral patterns, functional fluctuations, and early signs of change [19]. While such knowledge is often clinically meaningful, it is unevenly documented and therefore vulnerable to variation [20]. Differences in staff experience and training [21], challenges related to documenting unstructured information [22], and technical constraints in digital systems [23] may all contribute to variation in how observations are recorded. Consequently, clinically relevant information may remain undocumented or circulate primarily through informal communication. Structured, high-frequency observational routines may offer one way to stabilize and render this tacit knowledge more visible and actionable. In dementia care, key data types that benefit from systematic capture include the presence and severity of behavioral and psychological symptoms, daily functional fluctuations, and context cues such as activity, sleep, and interpersonal interactions. Documenting these systematically rather than informally is a prerequisite for using them in care planning.

### BPSD as a Case of Rapid Symptom Fluctuation

Dementia, and especially BPSD, provides a particularly relevant case for examining this documentation gap, as symptoms fluctuate episodically across time, environments, and internal states. Dementia is a progressive neurocognitive condition affecting a large and growing proportion of older adults worldwide [24,25], and up to 90% of people living with dementia experience BPSD over the course of the condition [26,27]. Symptoms include agitation, anxiety, hallucinations, delusions, irritability, apathy, and sleep disturbances. BPSD substantially affects quality of life, caregiver burden, rates of institutionalization, and the need for tailored interventions [28,29].

Moreover, several BPSD symptoms share overlapping behavioral expressions, making clinical differentiation challenging even for experienced staff [30]. This interpretive complexity adds a further dimension to the documentation challenge, as structured assessment depends not only on frequency of observation but also on the clarity with which symptoms can be distinguished and categorized.

Because BPSD emerges from dynamic interactions among neurocognitive decline, personal needs, and environmental context, it requires close and repeated observation. Yet current assessment practices rely largely on periodic evaluations, while day-to-day fluctuations are often documented informally or recalled from memory [31]. In Sweden, the national BPSD Registry provides a structured framework for assessment, but registrations typically occur at intervals of 6 months or more [32]. This structural mismatch between rapidly fluctuating symptoms and episodic assessment systems [33] may leave clinically meaningful variation undetected, limiting opportunities for timely and person-centered intervention.

One possible way to capture this variation is through structured high-frequency observational routines carried out as part of everyday care practice. This raises interrelated questions: whether such structured observation is feasible to perform at all and whether it can be integrated as a routine practice in residential dementia care. The latter is shaped by interrelated technical, interpretive, and organizational conditions. Before larger-scale adoption can be considered, it is therefore necessary to examine whether structured daily observational routines are feasible under real-world conditions [34].

### Objective

The overall aim of this pilot study was to investigate whether structured daily observation of BPSD, supported by digital documentation, can be incorporated into everyday residential dementia care. Two research questions were addressed: (RQ1) What is the feasibility of integrating structured daily observations into residential dementia care? (RQ2) What are the day-to-day and between-resident variations in BPSD registrations? Feasibility was examined with adherence to the registration routine as the primary feasibility outcome, and practicality, acceptability, and integration into existing workflows were examined descriptively.

## Methods

### Study Design

A convergent mixed methods design was used to examine whether structured daily observation of BPSD, supported by digital documentation, can be incorporated into everyday dementia care. Quantitative and qualitative data were collected concurrently, analyzed separately, and subsequently brought together as part of a structured data integration process, in accordance with established principles for convergent mixed methods research [35].

A convergent design was chosen because the 2 strands addressed the same research question (the feasibility of integrating structured daily observational routines into care) from complementary perspectives, with neither strand subordinate to the other. The quantitative data on adherence, registration patterns, and shift-registration time describe what was accomplished; the qualitative data on staff experiences describe how and under what conditions. A sequential design was not appropriate because neither strand needed to inform the other prospectively, and an embedded design was not appropriate because neither was secondary. Concurrent data collection during the same 90-day study window further supported a convergent approach.

Feasibility was conceptualized using the focus areas described by Bowen et al [36]; four areas were targeted in this pilot: implementation, practicality, acceptability, and integration. The primary feasibility outcome was adherence to the registration routine, operationalized as the proportion of expected item-level registrations completed. The remaining 3 dimensions were explored descriptively without prespecified thresholds. The other 4 Bowen focus areas (demand, adaptation, expansion, and limited-efficacy testing) were considered beyond the scope of this pilot.

In this study, two related but distinct concepts are used in their established implementation-science sense: *implementation* refers to the process of introducing the routine into care (operationalized in this study through adherence, the primary feasibility outcome); *integration* refers to the extent to which the routine fits within existing workflows.

### Ethical Considerations

This study was approved by the Swedish Ethical Review Authority (Dnr 2022-04509-01) under two specific conditions reflecting the cognitive vulnerability of the resident population. First, because residents could not reasonably be expected to assimilate written study information themselves, written information was directed to a next of kin, legal guardian, or trustee, who provided consent on the resident’s behalf and was instructed to consult the resident and convey the resident’s opportunity to object. Because residents could not reliably provide active assent, the procedure relied on this proxy consent together with the resident’s ongoing opportunity to object, a nonobjection rather than an active-assent model. Second, the structured daily observations imposed no incremental procedural burden: registrations were integrated into the documentation that care staff would otherwise perform informally during routine care, requiring no additional interaction with the resident. To minimize the handling of personal data, signed consent forms were collected and stored locally by participating unit managers and were never transferred to the research team; the team verified that consent had been completed before each resident was included.

Health care professionals were informed of the study by their unit managers but registered their interest in interview participation directly with the research team via an online form, after which a subset were randomly approached; oral informed consent was obtained prior to each interview. Use of the Daily-BPSD (Jönköping University) application required personal staff login to preserve accountability, in line with standard clinical documentation practice; upon export to the research dataset, staff identifiers were replaced with study codes (eg, “Staff Participant 3”), and the anonymized registration data were stored on secure servers at Jönköping University. Audio recordings and verbatim transcripts of interviews were stored on secure university servers, with the key linking interview codes to identifiable information held separately at Jönköping University. The processing of personal data rested on a public-interest legal basis under the EU General Data Protection Regulation, together with the study’s ethical approval.

### Settings and Participants

The study was conducted in dementia-specific residential care units within municipal long-term care facilities in southern Sweden. The 4 participating units were located in 2 municipalities and distributed across both urban and smaller community settings, with all 4 operated by their respective municipalities. According to Swedish national guidelines for dementia care, such units are designed to provide round-the-clock care for persons with dementia who require extensive support in daily activities and continuous supervision. Care is typically delivered by assistant nurses, with registered nurses available on a scheduled basis. Staff typically worked in rotating shifts, with day shifts (approximately 7 AM to 3 PM), evening shifts (approximately 3 PM to 9 PM), and night shifts (approximately 9 PM to 7 AM). Dementia-specific units are commonly organized into small-scale living environments, or wards, intended to support person-centered care [37]. Although a unit may comprise several wards, the study residents in each unit belonged to a single ward with a capacity of 8‐10 residents.

Residents were eligible if they lived in a designated dementia unit and had a medically confirmed diagnosis of dementia. No restrictions were applied regarding age or gender. All included residents exhibited BPSD, although no criteria were applied regarding specific symptoms or severity. Because many residents had significant cognitive impairment that limited their ability to provide informed consent, written proxy consent was obtained from legal representatives or next of kin.

All regular care staff working on the participating units were expected to contribute to the daily observational registrations during the study period as part of their regular work routines. Staff were eligible for qualitative interviews if they had been actively involved in the daily care of included residents.

Unit managers facilitated recruitment by informing staff about the study and coordinating contact with residents’ legal representatives or next of kin to obtain written proxy consent. Recruitment followed a convenience sampling approach appropriate for feasibility-oriented research. The sample of 8 residents reflected the practical capacity of the participating units and the research team to support intensive daily observation over the 90-day pilot. Residents were included from all 4 participating units so that the pilot captured daily documentation practices across each care context. Participation in the qualitative component was voluntary, and 10 assistant nurses consented to participate in interviews.

### Quantitative Data Collection

#### The Daily-BPSD Tool

Daily-BPSD is a web-based application designed to support structured daily observation of BPSD as part of everyday care routines. The tool enables staff to register symptom severity up to 3 times per day using smartphones, tablets, or computers.

**Figure 1.**
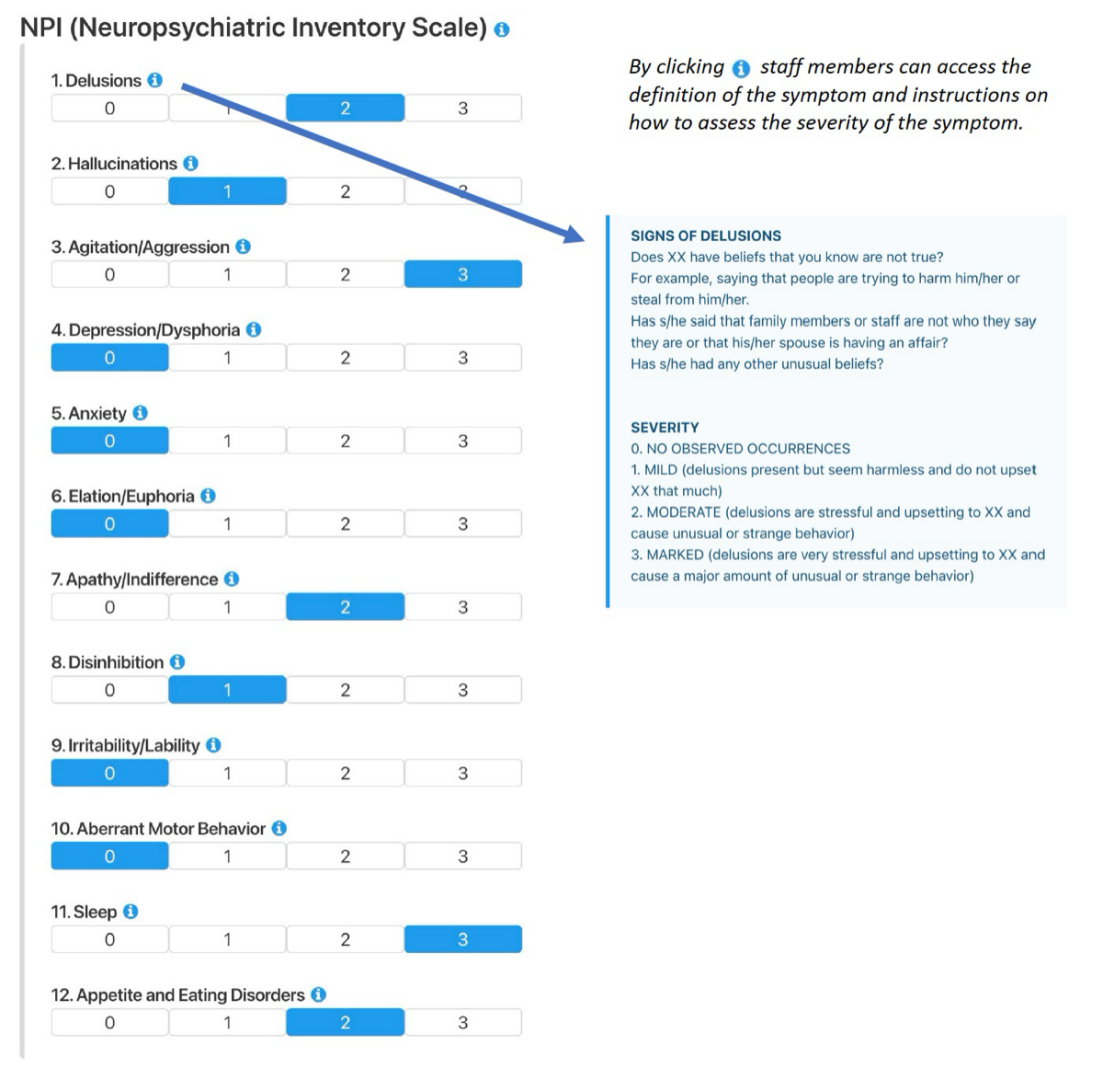
Staff interface for registering Neuropsychiatric Inventory (NPI) symptom severity in Daily-BPSD, including clickable definitions and standardized rating guidance. Screenshot from the Daily-BPSD website (2025 Jönköping University, Daily-BPSD project; reproduced with permission).

The interface is based on the 12 domains of the Neuropsychiatric Inventory (NPI) [38], with domain definitions aligned with the Neuropsychiatric Inventory – Nursing Home version (NPI-NH) [39]. Each domain includes a 0‐3 severity scale, supported by clickable definitions and examples, to promote consistent interpretation among staff (see Figure 1). In addition to the predefined NPI domains, the tool allows optional individualized variables to capture specific care needs or context-sensitive interventions.

Daily-BPSD automatically generates graphical summaries of symptom trends over time directly within the application as new observations are registered, supporting reflection and team communication. The tool was developed in collaboration with care staff and relatives and iteratively adapted to fit existing care workflows, informed by previous findings [31].

#### Quantitative Data Description

Prior to the study period, staff members in participating units received a 2-hour introduction to the Daily-BPSD application, conducted by members of the research team. The introduction covered the rationale for daily symptom registration, navigation of the registration interface, interpretation of NPI severity definitions via the in-app information texts (see Figure 1), and account login.

Using this tool, structured daily observations were recorded for 8 residents over a 90-day period. The system automatically time-stamped each entry, allowing analysis of adherence patterns and temporal distribution of observations. Data were exported in anonymized form and contained no personal identifiers beyond each resident’s study code. The quantitative dataset therefore captured both the volume and distribution of observational activity as well as symptom patterns across the study period.

Throughout this study, a *registration* (or *item-level registration*) refers to a single severity rating recorded for one item (either an NPI-NH symptom domain or an individually selected variable) for one resident at one shift; reported registration totals therefore reflect the number of item-level rating events. A *shift registration* refers to one completed NPI form for one resident at one shift (up to 3 per resident per day, one per shift); the 19,123 NPI item-level registrations were recorded across 1623 completed shift registrations. The 2870 individually selected variable registrations are item-level events not tied to a shift form. Registration time is reported per shift registration, not per item-level registration.

### Qualitative Data Collection

Qualitative data were collected through semistructured dyadic interviews with 10 assistant nurses conducted in 5 pairs. One pair was recruited from each of the 4 participating units, with one unit contributing an additional pair to include night shift staff. Dyadic interviews were used to encourage interaction between participants and to generate richer accounts of shared experiences within their common care context, allowing participants to validate, contrast, and elaborate on each other’s accounts in ways that solo interviews typically do not [40,41]. Participants were eligible if they were regular staff members working on the participating units during the quantitative data collection period and had used the Daily-BPSD tool for daily symptom observation. These 10 assistant nurses were drawn from the care staff who performed the daily observations and contributed to the study by sharing their experiences in the interviews analyzed below. The semistructured interview guide used during the interviews is provided in Multimedia Appendix 1.

The interviews were held at the end of the observation period to allow participants to reflect on their experiences throughout the monitoring period. The interview guide covered topics related to daily observational routines, how behavioral and psychological symptoms were interpreted, factors affecting symptom judgment, and contextual conditions influencing feasibility, such as workload, team coordination, and local documentation practices. Questions were informed by earlier developmental work [31] and by selected domains of the NASSS (nonadoption, abandonment, scale-up, spread, and sustainability) framework [42], which also guided the subsequent deductive analysis.

All interviews were conducted by members of the research team in the participating residential care units, audio recorded with consent, transcribed verbatim, and anonymized. Field notes were taken to capture situational aspects relevant for later analysis.

### Data Analysis

#### Analytic Framework

Whereas the feasibility outcomes specified above identified what was assessed in this study, the NASSS framework [42] was applied to interpret why feasibility-relevant patterns emerged. NASSS is widely used to examine the implementation, scale-up, and sustainability of health technologies in complex care systems [43-45]. Based on the study’s aim and familiarization with the empirical material, 5 NASSS domains were identified as analytically relevant: condition, technology, value proposition, adopters, and organization. To facilitate integration of qualitative findings with the quantitative data on adherence and symptom patterns, qualitative findings are reported under the NASSS domains used in the deductive analysis rather than independent categories.

The *organization* domain was included to account for unit-level variation in workflow, leadership support, staffing patterns, and local routines, all of which shaped the feasibility of integrating structured daily observational practices into everyday care.

The *condition* domain was particularly relevant because BPSD are fluctuating, context-dependent, and often unpredictable qualities that fundamentally shape what structured daily observational practices must capture.

The *technology* domain was used to examine the work required to conduct structured daily observations, including usability aspects of the digital interface and how the routine aligned with existing documentation practices.

The *value proposition* domain captured staff perceptions of the usefulness of frequent documentation for reflection, communication, care planning, and understanding residents’ needs.

The *adopters* domain addressed how staff members’ interpretive skills, motivation, confidence, and perceived workload influenced both the consistency with which observations were conducted and the extent to which the resulting data were used in practice.

These domains informed the deductive coding matrix in the qualitative analysis and provided a structured lens for integrating quantitative and qualitative findings across the convergent mixed methods design. While the NASSS domains provided the primary analytical structure, the analysis remained open to relevant insights not fully captured by the predefined domains. The remaining NASSS domains (wider system and embedding and adaptation over time) were not included because they address system-level influences and long-term adaptation processes that were beyond the scope of this study.

#### Quantitative Analysis

Quantitative data were analyzed descriptively to examine adherence to daily observational routines, the frequency and distribution of documented symptoms, and temporal variation across the observation period. For each resident, the total number of item-level registrations, the proportion of days with completed entries, and the number of contributing staff members were calculated. Symptom frequencies were summarized for each BPSD domain, and the distribution of daily symptom counts was examined to assess variation across residents. Time-stamped entries enabled analysis of registration frequency across shifts and adherence patterns across units. Because the study aimed to assess feasibility rather than effectiveness, inferential testing was limited to examining changes in the time taken to complete a shift registration. For each resident, shift-registration times (one completed shift-form, measured from form opening to submission) during the first (days 1‐30) and last (days 61‐90) 30-day intervals were compared using the Welch independent *t* test, which is robust when the variances are not equal [46]. Durations exceeding 5 minutes were treated as outliers and excluded (43 of 1623 shift registrations, 2.6%), as these likely reflected staff interruptions or forgotten submissions. All quantitative analyses were conducted in Python (version 3.12) using standard scientific computing libraries.

#### Qualitative Analysis

Qualitative data from dyadic interviews were analyzed using deductive qualitative content analysis following the approach described by Elo and Kyngäs [47]. The analysis proceeded in 3 stages: preparation, organizing, and reporting. In the preparation phase, transcripts were read repeatedly to obtain a comprehensive understanding of staff experiences. In the organizing phase, meaning units related to staff experiences of conducting daily symptom observations were identified and organized into a structured coding matrix informed by selected domains of the NASSS framework (see Multimedia Appendix 2 for an example).

Codes were iteratively compared, refined, and grouped into categories through discussions within the research team. In the reporting phase, these categories were organized within the corresponding NASSS domains, capturing staff perspectives on the feasibility, challenges, and perceived value of conducting daily symptom observations. Microsoft Excel (Microsoft 365 version) and Python (version 3.12) were used for data management, coding, and analysis. Coding was performed by JM. Each meaning unit was assigned a descriptive code that was then mapped to a generic category and to its corresponding NASSS domain; Multimedia Appendix 2 provides a worked example of this quote-to-code-to-category-to-domain chain. EO and TB reviewed a subset of coded segments and provided input on categorization, and points of uncertainty were discussed within the team until a shared interpretation was reached. To support the trustworthiness of the analysis, coding decisions were documented in the structured coding matrix and revisited iteratively in team discussions. We did not compute intercoder agreement statistics, as such measures are of limited interpretive value for deductive content analysis with categories prespecified from a theoretical framework.

#### Integration of Data

Following separate analyses of the 2 strands, integration was conducted in 3 procedural steps, following the joint-display approach described by Fetters et al [35]. First, findings from each strand were summarized within the 4 feasibility dimensions used in this study (implementation, practicality, acceptability, and integration). Second, quantitative and qualitative evidence pertaining to the same dimension were juxtaposed to identify points of convergence (where strands reinforced each other), complementarity (where strands addressed different facets of the same dimension), and divergence (where strands offered conflicting signals) [48]. Third, integrated meta-inferences were drawn within each NASSS domain to interpret why the observed feasibility patterns emerged, drawing jointly on registration data and staff accounts. A joint display summarizing the integrated evidence per feasibility dimension is provided in Multimedia Appendix 3.

## Results

### Participant Characteristics

Eight residents living with dementia and exhibiting symptoms of BPSD were included in the pilot study across 4 residential care units. Daily observations were conducted over a 90-day period using the Daily-BPSD tool. In total, 44 staff members contributed registrations to the system.

Table 1 presents residents’ age and gender, along with the number of staff members involved in observations for each resident. During the study period, care staff completed 1623 shift registrations (one per resident per shift; 75% of the 2160 possible resident-shifts, that is, 8 residents × 90 days × 3 shifts), comprising 19,123 NPI item-level ratings, together with 2870 individually selected variable registrations (21,993 item-level registrations in total; see Multimedia Appendix 4 for the full list).

**Table 1.** Characteristics of people living with BPSD^a^ residing in the participating residential care units, as well as the number of care staff using the tool during the data collection period.

Characteristics	Values
Residents, n	8
Age (years), mean (range)	84 (75‐92)
Gender, n (%)	
Female	5 (63)
Male	3 (37)
Care units, n	4
Unique staff members contributing registrations, n	44
Staff members per resident, median (range)	12 (7‐15)
Total registrations, n	21,993
NPI^b^ domain registrations, n	19,123
Individually selected variable registrations, n	2870

a BPSD: behavioral and psychological symptoms of dementia.

b NPI: Neuropsychiatric Inventory.

The deductive content analysis yielded 1137 coded segments across the 5 dyadic interviews. As shown in Table 2, the coding was distributed across all 5 NASSS domains, with the *adopters* and *organization* domains most prominently represented, followed by *technology*, *value proposition*, and the *condition*. The following sections present findings within each domain.

**Table 2.** Overview of NASSS^a^ framework coding across 5 interviews (N=1137 coded segments).

NASSS domain	Segments, n (%)	Generic categories
The Organization	319 (28.1)	Registration routine (n=130), workflow (n=118), team discussion (n=34), decision-making process (n=19), communication (n=9), scalability (n=7), the BPSD^b^ registry (n=2)
The Condition	75 (6.6)	Symptom presentation (n=41), symptom assessment (n=18), appropriate symptoms (n=10), dementia (n=6)
The Technology	231 (20.3)	Usability (n=96), registration device (n=42), information boxes (n=32), improvement suggestions (n=30), technical problems (n=25), zero function (n=5), missing data (n=1)
The Value Proposition	179 (15.7)	Perceived value (n=78), changed care practices (n=49), statistics use (n=42), data quality (n=10)
The Adopters	333 (29.3)	Competence (n=72), new employees (n=45), colleagues’ views (n=42), knowledge of residents (n=39), engagement (n=37), knowledge development (n=34), expectations (n=29), barriers (n=23), temporary staff (n=12)

a NASSS: nonadoption, abandonment, scale-up, spread, and sustainability framework.

b BPSD: behavioral and psychological symptoms of dementia.

### Domain: The Organization

Organizational routines shaped how Daily-BPSD registrations were carried out in practice. As context for the registration patterns presented below, day shifts typically comprised 3 staff members per unit, with 1 designated to complete the shift registration; these shift registrations were usually completed toward the end of the shift. Staff occasionally consulted colleagues to clarify observations for residents with whom they had less contact.

Units used different strategies to assign this responsibility. In some cases, it was integrated into existing routines by linking the task to other documentation responsibilities. As staff members described, *“*When we sit down to sign off medications and do our documentation, we added it as part of that routine as well” [Unit B, Staff Participant 3] and “Then we talk it through—the person on the green shift keeps track of the resident we’re registering, checks how the past day has been. And the others who worked the day also contribute, reflecting and choosing which ratings fit” [Unit C, Staff Participant 6], illustrating how registration responsibilities were integrated into established practical arrangements within the unit. At night, staff members sometimes worked alone while covering multiple wards, meaning they held overall responsibility for several residents simultaneously.

Staff emphasized the importance of ensuring that all team members were introduced to the tool and felt confident using it, so that the process would not depend on a few individuals. Several also emphasized the importance of initiating new cases directly within the application, without waiting for administrator support.

As shown in Table 3, registration adherence was high, exceeding 70% for all shifts, except for one unit (unit B) where adherence was significantly lower.

**Table 3.** Proportion of completed item-level NPI^a^ registrations per resident and shift type during the study, calculated as the proportion of expected item-level NPI registrations for each resident–shift-type combination (denominator: 12 NPI domains × 90 study days = 1080 expected item-level registrations per resident per shift type). Residents are coded by unit (A-D) and a sequential resident study number (1-8).

Resident	Morning, %	Evening, %	Night, %	Overall, %	Staff, n
A1	91	90	76	85	12
A2	92	91	80	88	12
B3	58	66	13	46	10
B4	43	47	10	33	8
C5	89	83	98	90	7
D6	87	87	87	87	15
D7	87	86	92	88	15
D8	83	84	87	85	15
Mean	79	77	68	75	12^b^

a NPI: Neuropsychiatric Inventory.

b Total unique staff members across all residents: 44.

Analysis of registration frequency was conducted to examine changes in the number of completed item-level NPI registrations over the study period. Item-level NPI registration counts were divided into 3 intervals based on the number of days since the first registration for each resident: days 1‐30 (n=6881), days 31‐60 (n=6607), and days 61‐90 (n=5635). The number of completed item-level registrations decreased in the final period compared to the earlier phases. The decline in the final interval coincided with the summer vacation period, when temporary staff without access to the tool were more common (see Strengths and Limitations).

### Domain: The Condition or Illness

**Figure 2.**
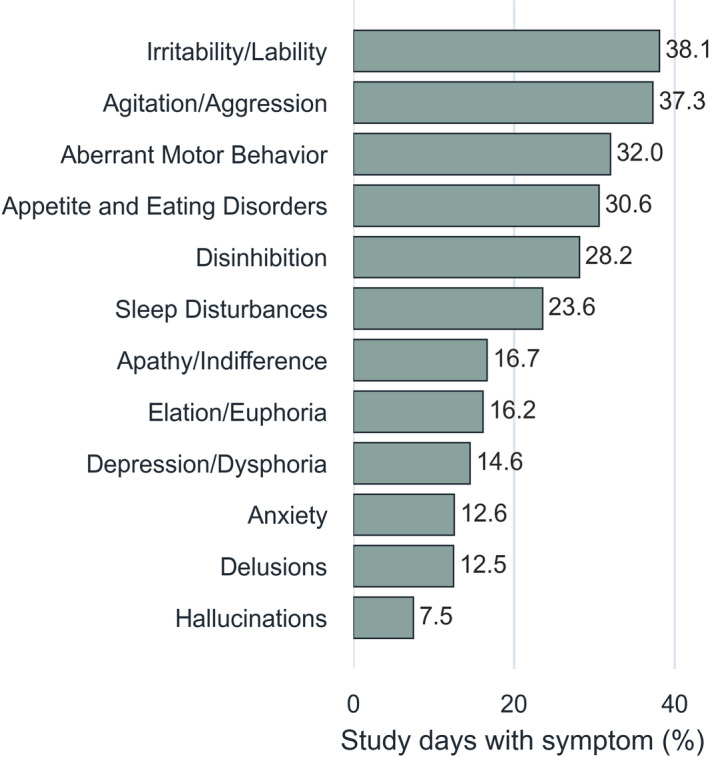
Proportion of observation days with recorded Neuropsychiatric Inventory (NPI) symptoms during the study period. Bars represent, for each symptom, the equal-weight mean across the 8 residents of the proportion of each resident’s observation days on which the symptom was recorded at least once (each resident contributing 52‐90 observation days).

The occurrence of BPSD symptoms varied across residents, both in terms of overall frequency and in the types of symptoms observed. As shown in Figure 2, the proportion of days with observed NPI symptoms differed between symptom domains. Across the study period, staff recorded symptom ratings (severity 0‐3) for the 8 included residents, who each contributed 52‐90 observation days (mean 82.1). Irritability/lability (38.1%) and agitation/aggression (37.3%) were the most frequently observed symptoms, indicating that these behaviors were present on more than one-third of all registered days and represented the most pervasive symptoms. The least frequently reported symptoms included *delusions* (12.5%) and *hallucinations* (7.5%), suggesting that psychotic features were relatively uncommon in daily care observations.

**Figure 3.**
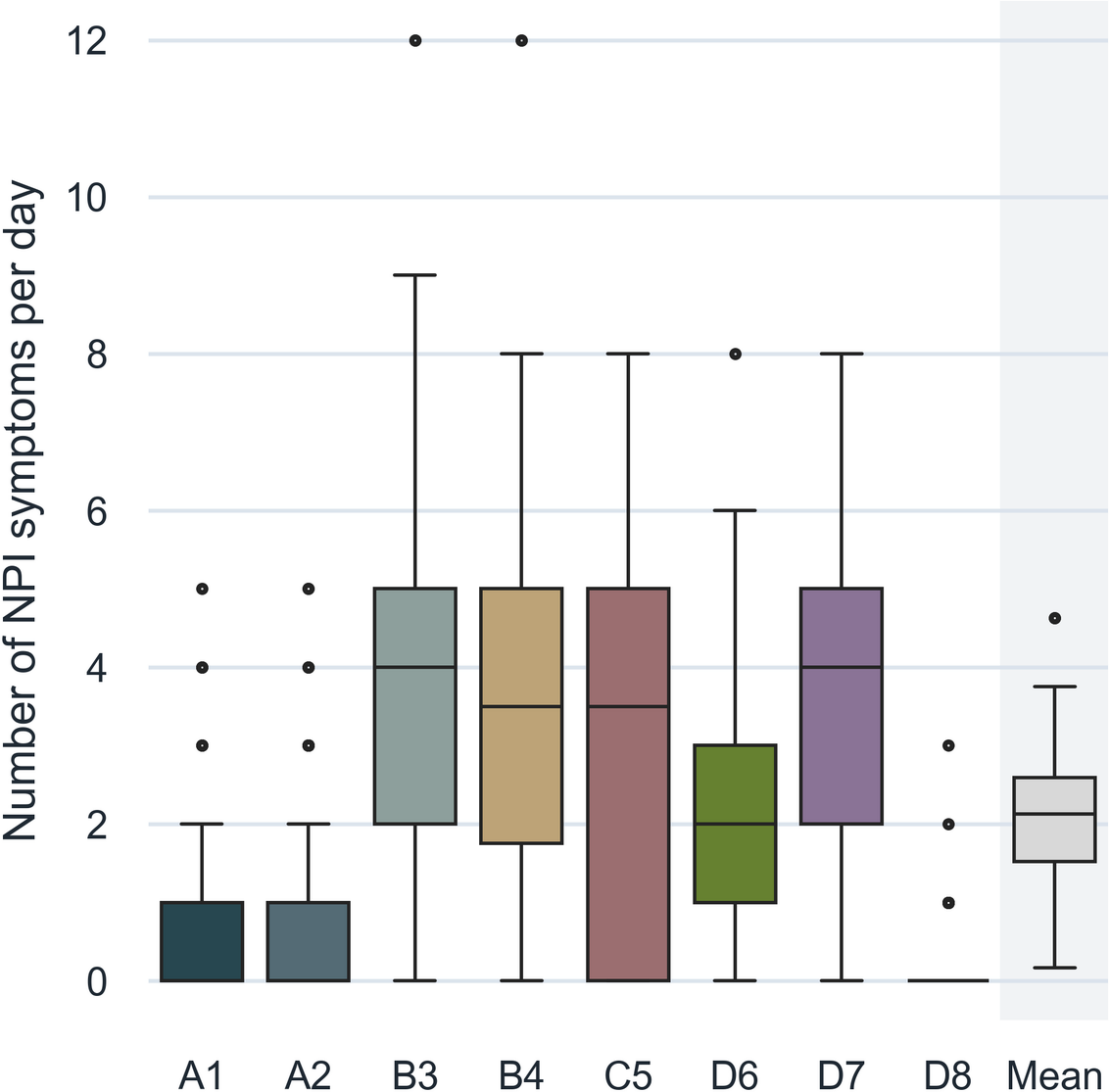
Number of reported Neuropsychiatric Inventory (NPI) symptoms per day across participants. Boxplots illustrate the distribution of daily recorded NPI symptoms for each resident across the 90-day study period. Each box represents IQR (25th-75th percentile), the horizontal line within the box indicates the median, whiskers extend to 1.5 × IQR, and dots represent outliers. The rightmost box “Mean (equal-weight)” shows the distribution of daily equal-weight means across all residents, where each resident contributes equally regardless of the number of registrations. Differences in medians and spread indicate variation in both overall symptom levels and day-to-day fluctuations among residents.

The number of symptoms recorded per day varied across residents during the study period. Median daily symptom counts ranged from 2 to 3 for most residents, although the spread within individuals varied substantially. Some residents showed relatively stable distributions with limited day-to-day variation, whereas others displayed wider distributions and occasional peaks with higher symptom counts (see Figure 3).

The nature of BPSD symptoms also shaped the interpretive demands placed on staff conducting daily observations. In the interviews, participants perceived certain symptoms, such as anxiety, hallucinations, and delusions, as particularly challenging to identify and assess in terms of severity. As one staff member reflected, “I think we’ve gotten better at distinguishing whether it’s a hallucination or a delusion […] Sometimes it’s a bit like, which is which, but then I find it helpful that you can read a little” [Unit D, Staff Participant 9]. The staff described that such situations often required close collaboration within the team to reach consensus and ensure reliable assessments. Knowing the individual resident was viewed as crucial for accurately interpreting symptoms, while temporary or new staff were said to struggle more with recognizing behavioral expressions correctly. Participants noted that the supportive texts (see Figure 1) integrated into the application were helpful when uncertainty arose. Although collective reflections based on group statistics were uncommon, participants reported that registering symptoms daily increased their confidence in assessing symptoms and provided better conditions for adjusting care to residents’ needs.

### Domain: The Technology

#### User Experience Feedback

Staff consistently described the Daily-BPSD tool as easy to use and time-efficient. The registration process was generally perceived as quick and straightforward, and few participants reported technical difficulties. As one participant explained, “It has been easy to log in, easy to fill in, and I think it doesn’t take very long” [Unit D, Staff Participant 7], reflecting the general perception that the tool could be integrated smoothly into daily routines. In the interviews, most staff described the smartphone as their most common device for completing registrations, with computers and tablets used occasionally. Night staff emphasized the usefulness of the existing feature, allowing all symptoms to be marked as “no observed occurrences” when a resident was asleep during the shift.

While the overall usability was rated positively, several participants noted areas where functionality could be improved. Some expressed a need for a clearer overview of remaining variables to register, as this occasionally led to omissions—particularly among the individually selected variables. Others highlighted that the information button linked to each symptom was highly appreciated, as it provided helpful definitions and guidance in moments of uncertainty. For future large-scale use, participants suggested adding administrative features to simplify the management of user accounts and the linking of individually selected variables to specific residents.

#### Registration Time

**Figure 4.**
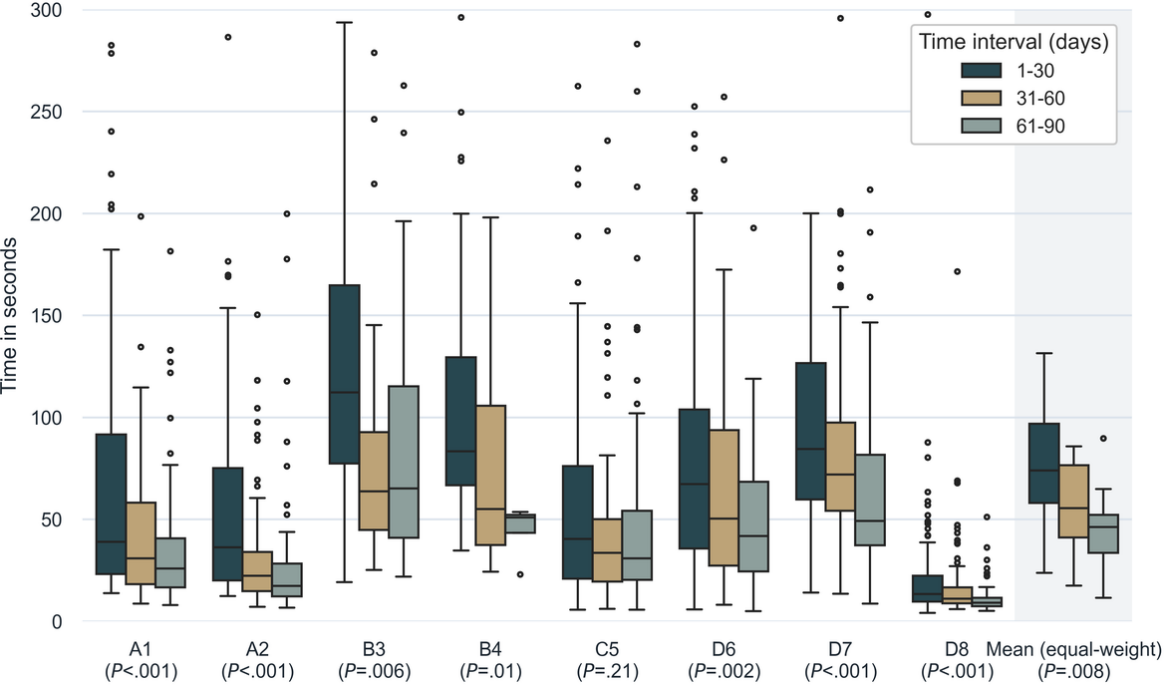
Average shift-registration time per participant across three 30-day intervals. Distribution of shift-registration times per resident during the observation period (days 1‐30, 31‐60, and 61‐90). Each box represents IQR (25th-75th percentile), the horizontal line within the box indicates the median, whiskers extend to 1.5 × IQR, and dots represent outliers. Color shading distinguishes the 3 time intervals. *P* values indicate Welch *t* test comparisons between the first and last interval. The category “Mean (equal-weight)” represents the distribution of daily means, with each resident weighted equally regardless of the number of registrations.

Consistent with staff perceptions that the tool was quick and easy to use, quantitative analysis of shift-registration times showed a reduction in the time required to complete a shift registration (measured from form opening to submission for one resident at one shift, ie, one complete shift-form) over the study period. Shift-registration times decreased significantly for all residents except C5 (*P*=.21) (see Figure 4). At the group level, the overall mean shift-registration time, calculated as an equal-weight average across residents (ie, each resident contributing equally regardless of the number of registrations), decreased from 77.1 seconds in the initial period to 46.2 seconds in the final period (*P*=.008), indicating that completing a full shift registration for all NPI domains together with any individually selected variables required less than 1 minute on average. This quantitative finding aligns with staff perceptions that the tool was quick to use in everyday practice.

### Domain: The Value Proposition

Participants described several ways in which daily symptom registration created value in their work. Staff reported that the process of assessing symptom severity on a daily basis increased their understanding of BPSD and strengthened their confidence in recognizing and discussing behavioral changes. As one participant expressed, “Then maybe you’ve understood that now she’s probably going to get angry, because you’ve seen that it happens every time, so maybe it’s washing her hands that triggers it” [Unit C, Staff Participant 6], illustrating how repeated symptom assessment was perceived as a learning process that increased staff awareness of behavioral changes. A reduction in average shift-registration time over the study period (see Figure 4) may also reflect increasing familiarity with both the tool and the symptom domains. Some participants also saw potential in using the aggregated data for team discussions. As one staff member noted, “I think that should have been the goal at team meetings, to bring up those statistics and see that he is much calmer when he has done this” [Unit A, Staff Participant 1]. However, only a few participants reported that the collected statistics had been actively used to adjust care for individual residents, although this was not an intended component of the pilot. Most participants nevertheless felt that the structured daily observational routine had clear potential to support both staff and residents if adopted more broadly in practice.

None of the participants raised economic issues, consistent with the study design, as they did not have financial responsibilities within their roles.

### Domain: The Adopters

**Figure 5.**
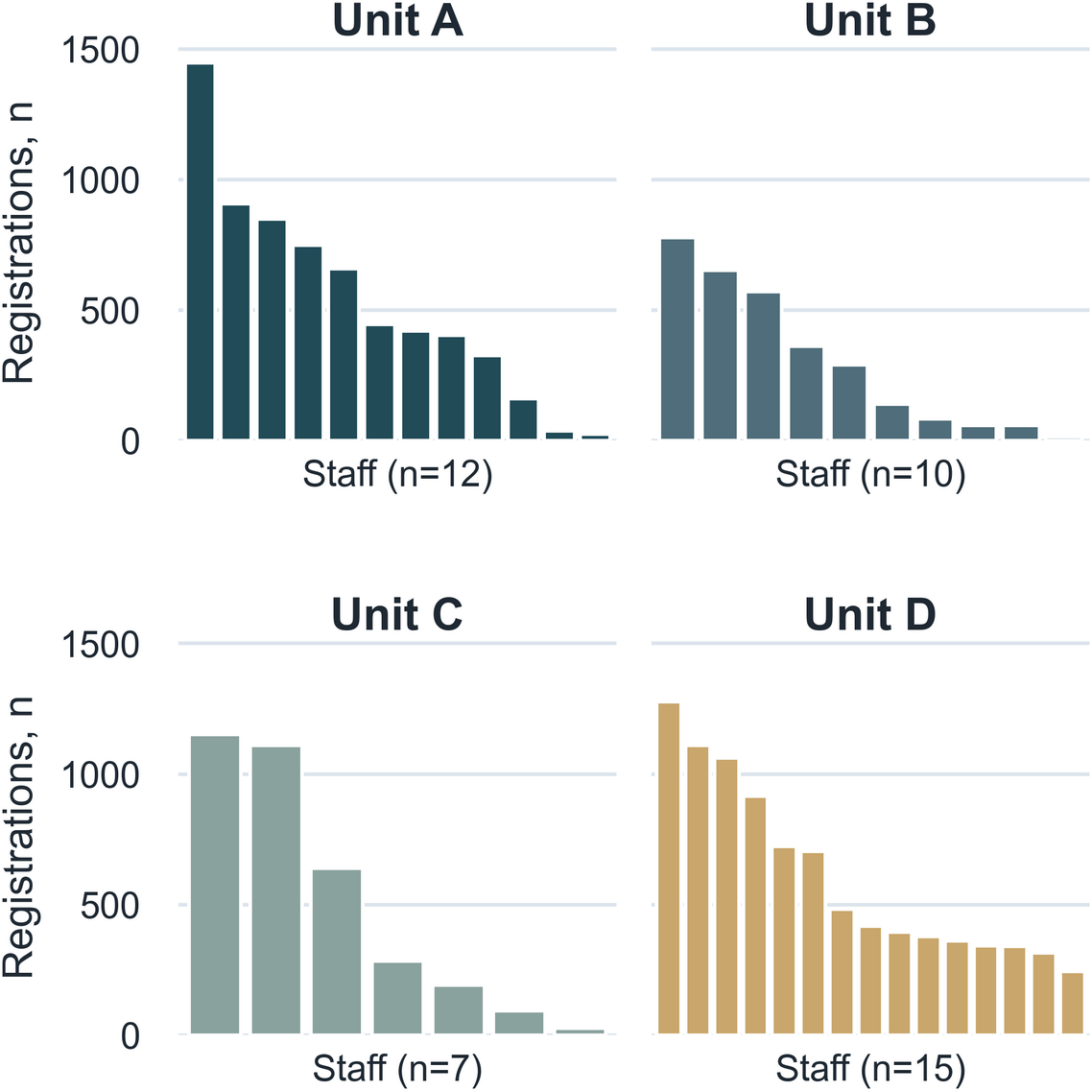
Number of Daily-BPSD item-level registrations per staff member across the 4 participating units. Each bar represents a unique staff member.

Participants emphasized the importance of engaging all staff members in the use of Daily-BPSD to ensure consistent registrations and shared ownership of the process. On some units, however, the registration effort was unevenly distributed: although all staff were expected to contribute, a few individuals carried out most of the registrations while others contributed comparatively little. As illustrated in Figure 5, the number of item-level registrations varied considerably among staff members within each unit, reflecting participants’ descriptions that some individuals took greater responsibility for completing Daily-BPSD registrations than others.

Participants also emphasized the importance of involving other health care professionals, such as nurses, occupational therapists, and physiotherapists, to improve the multidisciplinary value of Daily-BPSD. As one participant noted, *“*Personally, I think it’s better when everyone is involved. The more people you have, the more you can bounce ideas around. And then everyone is informed. Everyone has the same goal” [Unit C, Staff Participant 4]. Some noted that nurses currently rely on separate documentation systems, which limit information sharing and hinder the integration of Daily-BPSD data into broader care planning.

Some participants also expressed that Daily-BPSD might, in the long term, facilitate registrations in the national BPSD Registry by providing more structured, up-to-date information for use in formal assessments.

### Integrated Findings

Integration synthesized quantitative registration data with qualitative staff accounts within each of the 4 feasibility dimensions, characterizing each as showing convergence, complementarity, or divergence as defined in the Methods; a joint display summarizing the integrated evidence per dimension is provided in Multimedia Appendix 3.

For *implementation*, overall adherence of 75% varied substantially between units, with resident-level adherence ranging from 33% to 90% (Table 3). The qualitative data complemented this pattern by identifying two contrasting ways of organizing registration responsibility, as a shared task integrated into existing documentation routines, or as a task concentrated in a few individuals and therefore vulnerable to staffing disruptions, that plausibly underlie such between-unit variation.

For *practicality*, the reduction in shift-registration time from approximately 77 to 46 seconds (*P*=.008) converged with staff descriptions of the routine as quick, easy to log into, and naturally fitted to the closing routines of a shift.

For *acceptability*, quantitative adherence indicated *that* the routine was carried out, while qualitative data described *why* staff found it worth carrying out: reflection prompts, increased symptom awareness, and improved team conversations about residents. Complementarity dominated convergence here. A divergent signal also emerged: although staff perceived the routine as valuable, few reported that aggregated registration data had been used to actively adjust care during the pilot, suggesting that perceived value preceded actual use.

For *integration* into existing workflows, adherence differed markedly between units (Table 3). The qualitative accounts described how registration was fitted into a unit’s existing routines, by linking it to documentation or medication tasks and designating a staff member per shift to complete it, mechanisms that plausibly account for how readily the routine was absorbed into established workflows. Across the 4 dimensions, the convergent design surfaced not only points of agreement but also complementary and divergent signals that the quantitative data alone could not have revealed.

## Discussion

### Principal Findings

This pilot study examined the feasibility of integrating structured daily observational routines, supported by digital documentation, into residential dementia care, and also described the variation in BPSD registrations between residents and from day to day. Across the 4 feasibility dimensions, the primary outcome of *implementation* was supported by an overall adherence of 75% across shifts (range 33%‐90% between residents), indicating that structured daily symptom registration is achievable in routine care but exhibits substantial between-unit variation. *Practicality* was indicated by a reduction in shift-registration time from approximately 77 to 46 seconds over the 90-day period, consistent with staff accounts of the tool as quick and easy to use. *Acceptability* was reflected in staff descriptions of perceived value, increased symptom awareness, and meaningful contribution to their work. *Integration* into existing workflows differed between units, paralleling adherence patterns and reflecting differences in how registration responsibility was incorporated into established routines. Beyond feasibility, the data showed substantial variation in BPSD: NPI symptom prevalence ranged from 7.5% (hallucinations) to 38.1% (irritability/lability), and daily symptom counts varied both between and within residents, supporting the rationale for structured high-frequency observation as a means of capturing meaningful symptom variability.

A fundamental tension exists between the rapid fluctuation of BPSD and the episodic nature of current assessment systems. The strong adherence observed across most units shows that structured, frequent observational documentation does not conflict with routine dementia care, even in settings with workload and staffing challenges. The findings provide preliminary support for the argument that more frequent observational practices may help address the documentation gap between daily symptom dynamics and formal assessment systems [33,49].

At the same time, the decline in registrations during periods with increased temporary staffing highlights an important organizational vulnerability. Structured observational routines may be more sustainable when staff work within shared responsibilities and established workflows. In this study, some units integrated the registration task into collective routines, while others relied more on individual initiative. The considerable variation in individual registration activity within units suggests that integrating high-frequency documentation as a shared professional responsibility, rather than a task carried by a few committed individuals, remains a key implementation challenge in residential care settings.

The variation in completion rates also suggests that local organizational characteristics, rather than shift type alone, influenced adherence. Units with lower adherence, particularly during night shifts where staff sometimes worked alone across multiple wards, may require additional support to ensure consistency. This aligns with implementation research emphasizing that digital innovations become integrated into everyday workflows, through leadership engagement and ongoing reinforcement within teams [50,51].

The results also contribute to the discussion on tacit knowledge in long-term care. Staff described how conducting structured daily symptom assessments enhanced their awareness and reflective engagement with BPSD. This suggests that formalizing observational routines may not only stabilize documentation but also make previously tacit clinical judgments more explicit by encouraging staff to regularly articulate, compare, and reflect on their assessments of residents’ symptoms. At the same time, participants reported difficulties distinguishing between overlapping symptoms such as anxiety and agitation or hallucinations and delusions. Such challenges echo previous research demonstrating the interpretive complexity of BPSD assessment, even among experienced professionals [30,52]. Structuring observation does not eliminate interpretive uncertainty; rather, it makes it more visible and underscores the need for shared definitions and continuous training. Viewed through the NASSS framework, these findings illustrate how the adopter and condition domains interact: the interpretive complexity inherent in BPSD (condition) places demands on staff competence and confidence (adopters) that cannot be resolved by technical solutions alone.

Taken together, the findings indicate that when high-frequency data collection is applied to observation-based, subjectively interpreted phenomena, the conditions for feasibility differ from those of instrumental monitoring: sustainability was shaped less by technical infrastructure than by organizational continuity and shared professional practice. In this sense, structured daily observational documentation functions as a bridge between informal, experience-based knowledge and formalized assessment systems, and may complement existing quality registers by rendering day-to-day fluctuations more systematically visible without replacing professional judgment. Consistent with its feasibility focus, the study characterizes the conditions under which such documentation can be carried out but did not evaluate care outcomes: although staff reported increased symptom awareness and saw potential in using aggregated observations for team discussions, few had actively used the data to adjust care during the study period. Whether these conditions translate into more attentive care, improved symptom management, or resident-level outcomes was not assessed and requires direct evaluation in subsequent studies.

Future research should examine how such routines function across different organizational settings and over longer periods, the role of individualized staff-selected variables in capturing person-specific behavioral patterns, and how observational practices can be scaled and adapted across diverse care contexts.

### Strengths and Limitations

A key strength of this pilot study lies in its mixed methods design, which enabled a comprehensive examination of the feasibility and perceived value of structured, high-frequency observational documentation in dementia care. This design combined structured observational data on symptom patterns and adherence to the monitoring routine with qualitative accounts of staff experiences and the contextual conditions shaping its use.

However, several limitations should be acknowledged. The study involved a small, heterogeneous sample from a limited number of units in two municipalities in Sweden, limiting the transferability of the findings to other organizational contexts. Furthermore, the final study period (days 61‐90) coincided with the summer vacation period, during which a higher proportion of temporary staff, who lacked access to the tool, were on duty, likely contributing to the observed decline in registrations. This seasonal effect should be considered when interpreting adherence patterns. Variation in adoption patterns among staff members also indicates that unit-level feasibility does not necessarily imply uniform individual-level engagement.

The absence of resident-reported outcomes limits conclusions regarding the experiential impact of structured documentation on person-centered care. Furthermore, the relatively short 90-day follow-up period restricts assessment of long-term sustainability and penetration—key implementation outcomes in digital health research. As a pilot study, the findings primarily inform feasibility rather than effectiveness.

Despite these limitations, the study provides preliminary empirical support for the feasibility of integrating structured daily observational routines into residential dementia care and offers a foundation for further refinement and evaluation in larger-scale studies.

### Conclusions

This pilot study demonstrates that structured high-frequency observational documentation can be feasibly integrated into residential dementia care when supported by clear routines and team continuity. Integrating such practices into everyday work can establish a structured basis for systematic observation and team communication; whether this translates into earlier detection of behavioral changes or improved care quality requires further evaluation in studies designed to assess clinical and person-level outcomes.

The findings highlight that successful implementation depends less on technical functionality and more on organizational readiness, staff competence, and time allocated for reflection. Although Daily-BPSD served as the empirical platform in this study, the broader contribution concerns the feasibility of structured daily symptom documentation as a complement to existing quality registers. Such approaches may provide a more frequent and detailed informational basis for ongoing care processes; whether this can support more proactive and person-centered dementia care in practice remains an empirical question for future research.

## Supplementary material

10.2196/98024 Multimedia Appendix 1 Semistructured interview guide used for the dyadic interviews with care staff.

10.2196/98024 Multimedia Appendix 2 Example of the NASSS-guided coding matrix, showing coded segments across the 5 selected domains. NASSS: nonadoption, abandonment, scale-up, spread, and sustainability.

10.2196/98024 Multimedia Appendix 3 Joint display of integrated quantitative and qualitative evidence per feasibility dimension.

10.2196/98024 Multimedia Appendix 4 List of individually selected variables used in addition to the standard Neuropsychiatric Inventory domains in Daily-BPSD.

## Abbreviations

BPSD: behavioral and psychological symptoms of dementia
NASSS: nonadoption, abandonment, scale-up, spread, and sustainability
NPI: Neuropsychiatric Inventory
NPI-NH: Neuropsychiatric Inventory – Nursing Home version

## References

[1] Jones CH, Dolsten M (2024). Healthcare on the brink: navigating the challenges of an aging society in the United States. NPJ Aging.

[2] Tynkkynen LK, Pulkki J, Tervonen-Gonçalves L, Schön P, Burström B, Keskimäki I (2022). Health system reforms and the needs of the ageing population – an analysis of recent policy paths and reform trends in Finland and Sweden. Eur J Ageing.

[3] Ogugua JO, Muonde M, Maduka CP, Olorunsogo TO, Omotayo O (2024). Demographic shifts and healthcare: a review of aging populations and systemic challenges. Int J Sci Res Arch.

[4] Khan HTA, Addo KM, Findlay H (2024). Public health challenges and responses to the growing ageing populations. Public Health Chall.

[5] Ekman I, Swedberg K, Taft C (2011). Person-centered care – ready for prime time. Eur J Cardiovasc Nurs.

[6] Rosengren K, Brannefors P, Carlstrom E (2021). Adoption of the concept of person-centred care into discourse in Europe: a systematic literature review. J Health Organ Manag.

[7] McArthur C, Bai Y, Hewston P, Giangregorio L, Straus SE, Papaioannou A (2021). Barriers and facilitators to implementing evidence-based guidelines in long-term care: a qualitative evidence synthesis. Implementation Sci.

[8] Moore L, Britten N, Lydahl D, Naldemirci Ö, Elam M, Wolf A (2017). Barriers and facilitators to the implementation of person-centred care in different healthcare contexts. Scand J Caring Sci.

[9] Gopal G, Suter-Crazzolara C, Toldo L, Eberhardt W (2019). Digital transformation in healthcare – architectures of present and future information technologies. Clin Chem Lab Med.

[10] Mitchell M, Kan L (2019). Digital technology and the future of health systems. Health Syst Reform.

[11] Pyper E, McKeown S, Hartmann-Boyce J, Powell J (2023). Digital health technology for real-world clinical outcome measurement using patient-generated data: systematic scoping review. J Med Internet Res.

[12] Maiorino MI, Signoriello S, Maio A (2020). Effects of continuous glucose monitoring on metrics of glycemic control in diabetes: a systematic review with meta-analysis of randomized controlled trials. Diabetes Care.

[13] Campanella G, Hanna MG, Geneslaw L (2019). Clinical-grade computational pathology using weakly supervised deep learning on whole slide images. Nat Med.

[14] McKinney SM, Sieniek M, Godbole V (2020). International evaluation of an AI system for breast cancer screening. Nature.

[15] Connor J, Flenady T, Massey D, Dwyer T (2023). Clinical judgement in nursing – an evolutionary concept analysis. J Clin Nurs.

[16] Casey AN, Low LF, Goodenough B, Fletcher J, Brodaty H (2014). Computer-assisted direct observation of behavioral agitation, engagement, and affect in long-term care residents. J Am Med Dir Assoc.

[17] Powers JH, Patrick DL, Walton MK (2017). Clinician-reported outcome assessments of treatment benefit: report of the ISPOR Clinical Outcome Assessment Emerging Good Practices Task Force. Value Health.

[18] Beattie Z, Miller LM, Almirola C (2020). The collaborative aging research using technology initiative: an open, sharable, technology-agnostic platform for the research community. Digit Biomark.

[19] Laukvik LB, Lyngstad M, Rotegård AK, Slettebø Å, Fossum M (2022). Content and comprehensiveness in the nursing documentation for residents in long-term dementia care: a retrospective chart review. BMC Nurs.

[20] Eikelboom WS, Koch J, Beattie E (2023). Residential aged care staff perceptions and responses towards neuropsychiatric symptoms: a mixed methods analysis of electronic healthcare records. Aging Ment Health.

[21] Tshering G, Troeung L, Walton R, Martini A (2024). Factors impacting clinical data and documentation quality in Australian aged care and disability services: a user-centred perspective. BMC Geriatr.

[22] Larjow E, von Fintel M, Busse A (2022). A mixed-methods study of quality differences between applied documentation approaches in nursing homes. BMC Nurs.

[23] Kariotis TC, Prictor M, Chang S, Gray K (2022). Impact of electronic health records on information practices in mental health contexts: scoping review. J Med Internet Res.

[24] Nichols E, Steinmetz JD, Vollset SE (2022). Estimation of the global prevalence of dementia in 2019 and forecasted prevalence in 2050: an analysis for the Global Burden of Disease Study 2019. Lancet Public Health.

[25] (2025). Dementia, fact sheet. World Health Organization.

[26] Cerejeira J, Lagarto L, Mukaetova-Ladinska EB (2012). Behavioral and psychological symptoms of dementia. Front Neurol.

[27] Laganà V, Bruno F, Altomari N (2022). Neuropsychiatric or behavioral and psychological symptoms of dementia (BPSD): focus on prevalence and natural history in Alzheimer’s disease and frontotemporal dementia. Front Neurol.

[28] Górska S, Forsyth K, Maciver D (2018). Living with dementia: a meta-synthesis of qualitative research on the lived experience. Gerontologist.

[29] Savva GM, Zaccai J, Matthews FE (2009). Prevalence, correlates and course of behavioural and psychological symptoms of dementia in the population. Br J Psychiatry.

[30] Melander C, Sävenstedt S, Olsson M, Wälivaara BM (2018). Assessing BPSD with the support of the NPI-NH: a discourse analysis of clinical reasoning. Int Psychogeriatr.

[31] Malm J, Odzakovic E, Kåreholt I, Fristedt S, Bielsten T (2026). Exploring conditions for implementing daily monitoring of behavioral and psychological symptoms of dementia (BPSD) in dementia care: insights from a participatory design study. BMC Geriatr.

[32] Jönsson L, Wibom M, Londos E, Nägga K (2025). Person‐centered care at population scale: the Swedish Registry for Behavioral and Psychological Symptoms of Dementia. Alzheimers Dement Transl Res Clin Interv.

[33] Fauth EB, Gibbons A (2014). Which behavioral and psychological symptoms of dementia are the most problematic? Variability by prevalence, intensity, distress ratings, and associations with caregiver depressive symptoms. Int J Geriatr Psychiatry.

[34] Thabane L, Ma J, Chu R (2010). A tutorial on pilot studies: the what, why and how. BMC Med Res Methodol.

[35] Fetters MD, Curry LA, Creswell JW (2013). Achieving integration in mixed methods designs – principles and practices. Health Serv Res.

[36] Bowen DJ, Kreuter M, Spring B (2009). How we design feasibility studies. Am J Prev Med.

[37] (2017). Nationella riktlinjer för vård och omsorg vid demenssjukdom: Stöd för styrning och ledning [in Swedish]. https://www.socialstyrelsen.se/contentassets/8315c1b65e064fcb92065392c74b942c/2017-12-2.pdf.

[38] Cummings JL, Mega M, Gray K, Rosenberg-Thompson S, Carusi DA, Gornbein J (1994). The Neuropsychiatric Inventory: comprehensive assessment of psychopathology in dementia. Neurology.

[39] Cummings JL (2009). Neuropsychiatric Inventory – Nursing Home Version (NPI-NH). https://www.dementiaresearch.org.au/wp-content/uploads/2016/01/NPI-NH_cr.pdf.

[40] Eisikovits Z, Koren C (2010). Approaches to and outcomes of dyadic interview analysis. Qual Health Res.

[41] Morgan DL, Ataie J, Carder P, Hoffman K (2013). Introducing dyadic interviews as a method for collecting qualitative data. Qual Health Res.

[42] Greenhalgh T, Wherton J, Papoutsi C (2017). Beyond adoption: a new framework for theorizing and evaluating nonadoption, abandonment, and challenges to the scale-up, spread, and sustainability of health and care technologies. J Med Internet Res.

[43] Abell B, Naicker S, Rodwell D (2023). Identifying barriers and facilitators to successful implementation of computerized clinical decision support systems in hospitals: a NASSS framework-informed scoping review. Implement Sci.

[44] James HM, Papoutsi C, Wherton J, Greenhalgh T, Shaw SE (2021). Spread, scale-up, and sustainability of video consulting in health care: systematic review and synthesis guided by the NASSS framework. J Med Internet Res.

[45] Kadesjö Banck J, Bernhardsson S (2020). Experiences from implementation of internet-delivered cognitive behaviour therapy for insomnia in psychiatric health care: a qualitative study applying the NASSS framework. BMC Health Serv Res.

[46] Derrick B, White P (2016). Why Welch’s test is type I error robust. Quant Methods Psychol.

[47] Elo S, Kyngäs H (2008). The qualitative content analysis process. J Adv Nurs.

[48] Schoonenboom J, Johnson RB (2017). How to construct a mixed methods research design. Köln Z Soziol.

[49] Lee KH, Lee JY, Kim B (2022). Person-centered care in persons living with dementia: a systematic review and meta-analysis. Gerontologist.

[50] Cresswell K, Sheikh A (2013). Organizational issues in the implementation and adoption of health information technology innovations: an interpretative review. Int J Med Inform.

[51] Dugstad J, Eide T, Nilsen ER, Eide H (2019). Towards successful digital transformation through co-creation: a longitudinal study of a four-year implementation of digital monitoring technology in residential care for persons with dementia. BMC Health Serv Res.

[52] Chan DKY, Chan LKM, Kuang YM, Le MNV, Celler B (2022). Digital care technologies in people with dementia living in long-term care facilities to prevent falls and manage behavioural and psychological symptoms of dementia: a systematic review. Eur J Ageing.

