# Supplementary material for: Daily Monitoring of Behavioral and Psychological Symptoms of Dementia in Residential Care: Mixed Methods Pilot Study
Source: JMIR Aging. 2026 Jul 23;9:e98024. doi: 10.2196/98024 (PMC13395431; doi:10.2196/98024)
Supplement: Multimedia Appendix 1 [file aging-v9-e98024-s001.pdf]

Multimedia Appendix 1 – Interview Guide

The table below presents the semi-structured interview guide used in the dyadic interviews with care staff who used the Daily-BPSD application during the pilot study.

Main Questions	Focus/Follow-up Questions
1. Registration routine	
• Can you describe how you carried out the registrations?<br><br>• Can you tell me about the process of deciding who among you would do the registrations?<br><br>• How did you discuss the registrations with other assistant nurses or care staff in your team?<br><br>• Have you made any changes to your registration routine during the study? If so, can you describe what was changed?	• Who was responsible for the registrations?<br><br>• When were the registrations made?<br><br>• What type of device was used (mobile, computer, tablet)?<br><br>• How was agreement or consistency between staff handled?
2. The application	
• Can you describe what it was like to use the application?<br><br>• Now that you have used the application, can you share any thoughts about how it could be improved or changed?	• Barriers and enablers – user-friendliness/technology<br><br>• Challenges or unclear functions in the application?
3. Follow-up	
• In the application there is a tab called “Statistics.” If you have used it, can you describe how you used it?<br><br>• Did you make any registrations in the national BPSD Registry while using Daily-BPSD? If so, how did you use that data?	• If you looked at the Statistics tab – why and in what context?<br><br>• Did you view the statistics together as a staff group? Was the unit manager involved?
4. Inclusion	
• Can you reflect on whether Daily-BPSD could help people with BPSD?<br><br>• Assuming that Daily-BPSD could be helpful, who should decide if it is suitable for a particular person?<br><br>• If Daily-BPSD could add value and you identified that a person might benefit, what support or help would you need to start using it for each new case?	• When? Which residents?<br><br>• What criteria or symptoms are appropriate as a starting point?<br><br>• How could inclusion take place?<br><br>• Would external expertise be needed to get started, and if so, in which parts of the inclusion process?
5. BPSD symptoms	
• Can you tell me if there were any challenges in determining whether a person was showing BPSD symptoms?	• Challenges due to lack of knowledge about BPSD symptoms?

Main Questions	Focus/Follow-up Questions
• Can you tell me if using Daily-BPSD has affected your knowledge about the different BPSD symptoms?	
6. Behavioral changes	
• Can you describe if you made any changes during the study period in relation to using Daily-BPSD?<br><br>• Can you describe if you made any changes in daily care for the persons with BPSD because of using Daily-BPSD?<br><br>• Can you tell me if any organizational changes occurred with the introduction of Daily-BPSD? If so, which ones?	• Changes in registration routines?<br><br>• Work-related changes?<br><br>• Care-related changes? Other?
7. Concluding questions	
• Do your experiences of using Daily-BPSD correspond to the expectations you had before starting the registrations?<br><br>• A larger test of Daily-BPSD will begin in the autumn and continue for two years. What do you think is important to consider to make Daily-BPSD work and be helpful for persons with BPSD?<br><br>• Is there anything else you would like to add or tell us about your experiences of using Daily-BPSD?	• User-friendliness? Usefulness? Training needs?
General follow-up questions	
• Can you tell me more?<br><br>• What do you think this depends on?<br><br>• In what way?<br><br>• Can you give an example?<br><br>• How did you do then?<br><br>• Can you describe a specific situation where it worked really well?<br><br>• Can you describe a specific situation that was challenging and why?	
