# Supplementary material for: Daily Monitoring of Behavioral and Psychological Symptoms of Dementia in Residential Care: Mixed Methods Pilot Study
Source: JMIR Aging. 2026 Jul 23;9:e98024. doi: 10.2196/98024 (PMC13395431; doi:10.2196/98024)
Supplement: Multimedia Appendix 2 [file aging-v9-e98024-s002.pdf]

Multimedia Appendix 2 – Example of the NASSS-guided coding matrix

The table below shows a worked example of the NASSS-guided deductive coding matrix, illustrating the chain from interview quote to descriptive code to generic category to NASSS domain.

**Quote**	**Code**	**Generic Categories**	**Main Category (NASSS Domain)**
Because then you get a daily picture of how things have been—some days you're off and then you have no idea what it looked like.	Daily registration compensates for gaps in staff continuity	Workflow	The Organization
Information boxes appreciated as decision support during registration	Information boxes appreciated as decision support during registration	Information boxes	The technology
You start working differently—you get a completely different perspective on it all and a deeper understanding of why they act the way they do.	Registration changed staff's overall approach and understanding of residents	Perceived value	The Value Proposition
Personally, I think it's better when everyone is involved. The more people you have, the more you can bounce ideas around. And then everyone is informed. Everyone has the same goal.	Importance of collective engagement and shared ownership	Colleagues' perspectives	The Adopters
And if they don't eat, the anxiety and irritation become significant too.	Staff recognized interconnections between physical needs and BPSD symptoms	Symptom presentation	The Condition
