# Supplementary material for: Daily Monitoring of Behavioral and Psychological Symptoms of Dementia in Residential Care: Mixed Methods Pilot Study
Source: JMIR Aging. 2026 Jul 23;9:e98024. doi: 10.2196/98024 (PMC13395431; doi:10.2196/98024)
Supplement: Multimedia Appendix 3 [file aging-v9-e98024-s003.pdf]

Multimedia Appendix 3 – Joint display of integrated quantitative and qualitative evidence per feasibility dimension

Feasibility was operationalized through four focus areas from Bowen et al. [36]: implementation, practicality, acceptability, and integration. The convergent mixed-methods design integrated quantitative registration data with qualitative staff accounts within each dimension. The table below presents the integrated evidence side by side, with each row indicating points of convergence, complementarity, or divergence between the two data strands.

Feasibility dimension	Quantitative evidence	Qualitative evidence	Integrated interpretation
**Implementation** (primary feasibility outcome)	Overall adherence 75% across shifts (range 33–90% between residents; 68–79% across shifts). Substantial between-unit variation (Table 3).	Staff accounts of how registration responsibility was organized within the unit; descriptions of two contrasting arrangements, a shared task integrated into existing documentation routines, or a task concentrated in a few individuals and vulnerable to staffing disruption (Organization, Adopters).	**Complementary.** The qualitative accounts identify contrasting organizational arrangements that plausibly underlie the marked between-unit variation in adherence, without a one-to-one mapping of specific accounts to individual units.
**Practicality**	Mean shift-registration time decreased from ~77 s to ~46 s over 90 days (P = .008, equal-weight mean across residents; Figure 4).	Staff descriptions of the tool as quick, easy to log into, and naturally fitted to end-of-shift routines (Technology). Information buttons described as helpful when uncertainty arose.	**Strongly convergent.** Both strands indicate low time burden and good fit with existing workflow; quantitative trend matches staff perception of growing familiarity.
**Acceptability**	Continued use over 90 days at high overall adherence. No formal acceptability metric collected.	Staff accounts of perceived value, increased symptom awareness, meaningful contribution to their work, and improved team conversations about residents (Value Proposition, Adopters).	**Complementary** (dominant): quantitative shows *that* the routine was carried out; qualitative explains *why* it was perceived as worth doing. **Divergent signal**: although staff perceived the routine as valuable, few reported that aggregated registration data had been used to adjust care during the pilot, suggesting that perceived value preceded actual data-driven use.
**Integration** (Bowen dimension; distinct from data-strand integration)	Adherence varied markedly between units (Table 3).	Staff accounts of how registration was fitted into existing routines: linking it to documentation or medication tasks and designating a staff member per shift to complete it (Organization).	**Complementary.** The qualitative accounts describe organizational mechanisms that plausibly account for how readily the routine was absorbed into a unit's established workflow, helping interpret the between-unit variation in adherence without mapping specific accounts to individual units.
