# Supplementary material for: Daily Monitoring of Behavioral and Psychological Symptoms of Dementia in Residential Care: Mixed Methods Pilot Study
Source: JMIR Aging. 2026 Jul 23;9:e98024. doi: 10.2196/98024 (PMC13395431; doi:10.2196/98024)
Supplement: Multimedia Appendix 4 [file aging-v9-e98024-s004.pdf]

Multimedia Appendix 4 – List of Individually Selected Variables in Daily-BPSD

All registrations presented in this appendix are item-level registrations of individually selected variables that were added by care staff during the pilot study. In total, 21,993 item-level registrations were completed in the Daily-BPSD application, of which 2,870 corresponded to these individually selected variables. Eighteen unique variables were created, most of which included subdivisions that allowed differentiation between work shifts (e.g., morning, day, or night), corresponding to the staff members' respective registration periods. The table below presents all individually selected variables in alphabetical order, including the total number of registrations for each.

Variable	No. of Registrations
Activities	146
Amount of food (all meals)	477
Awake occasions observed	229
Communication problems	145
Creative activities	130
Daytime sleep (combined)	263
Home stay/visits	219
Meals (general)	170
Nicotine use during night	6
P-Glucose	6
PRN* medication	204
PRN* sedative and effect	5
Reaction during catheter change	66
Restless or unusual social interactions	293
Social activities	284
Time spent outdoors / walks outside unit	142
Uncooperative / accepting help	79
Unusual eating during night	6

* PRN (pro re nata) medication refers to medicine administered on an as-needed basis in response to acute symptoms, rather than on a fixed schedule.
